# Accuracy of the Delirium Observational Screening Scale (DOS) as a screening tool for delirium in patients with advanced cancer

**DOI:** 10.1186/s12885-019-5351-8

**Published:** 2019-02-19

**Authors:** Elisabeth C. W. Neefjes, Maurice J. D. L. van der Vorst, Manon S. A. Boddaert, Bea A. T. T. Verdegaal, Aart Beeker, Saskia C. C. Teunissen, Aartjan T. F. Beekman, Wouter W. A. Zuurmond, Johannes Berkhof, Henk M. W. Verheul

**Affiliations:** 1 Department of Medical Oncology, Cancer Center Amsterdam, Amsterdam UMC, Vrije Universiteit, Amsterdam, the Netherlands; 2 Department of Internal Medicine, Rijnstate Hospital, Arnhem, the Netherlands; 3 Netherlands Comprehensive Cancer Organisation (IKNL), Utrecht, the Netherlands; 4 Department of Internal Medicine, Spaarne Gasthuis, Hoofddorp, The Netherlands; 5 Department of General Practice, Julius Center for Health Sciences and Primary Care, University Medical Center Utrecht, Utrecht, the Netherlands; 6 Academic Hospice Demeter, De Bilt, The Netherlands; 7 Department of Psychiatry, Amsterdam UMC, Vrije Universiteit, De Bilt, The Netherlands; 8 Department of Anesthesiology, Amsterdam UMC, Vrije Universiteit, Amsterdam, the Netherlands; 9 Hospice Kuria, Amsterdam, The Netherlands; 10 Department of Epidemiology and Biostatistics, Amsterdam UMC, Vrije Universiteit, Amsterdam, the Netherlands; 11 Department of Medical Oncology, Cancer Center Amsterdam, Amsterdam UMC, Vrije Universiteit, De Boelelaan 1117, Rm 3A46, Amsterdam, 1081 HV the Netherlands

**Keywords:** Delirium, Diagnosis, Validation studies, Neoplasms, Palliative care

## Abstract

**Background:**

The Delirium Observation Screening Scale (DOS) was developed to facilitate early recognition of delirium by nurses during routine clinical care. It has shown good validity in a variety of patient populations, but has not yet been validated in hospitalized patients with advanced cancer, although the DOS is commonly used in this setting in daily practice. The aim of this study was to evaluate the accuracy of the DOS in hospitalized patients with advanced cancer using the revised version of the Delirium Rating Scale (DRS-R^− 98^) as the gold standard.

**Methods:**

Patients with advanced cancer admitted to the medical oncology ward were screened for delirium with the DOS and DRS-R−98. Sensitivity, specificity, negative predictive value (NPV) and positive predictive value (PPV) of the DOS were calculated, using a DOS score ≥ 3 as a cut-off for delirium.

**Results:**

Ninety-five DOS negative and 98 DOS positive patients were identified. Sensitivity of the DOS, was > 99.9% (95%-CI, 95.8–100.0%), specificity was 99.5% (95%-CI 95.5–99.96%), PPV was 94.6% (95% CI 88.0–97.7), and NPV was > 99.9% (95% CI 96.1–100.0).

**Conclusions:**

The DOS is an accurate screening tool for delirium in patients with advanced cancer. Since it has the benefit of being easily implicated in daily practice, we recommend to educate caregivers to screen patients with advanced cancer by DOS analysis. By early recognition and adequate treatment of this distressing delirium syndrome the quality of life of patients with advanced cancer can be improved.

**Trial registration:**

ClinicalTrials.gov Identifier NCT01539733 (Feb 27, 2012 - retrospectively registered), Netherlands Trial Register NTR2559 (Oct 7, 2010).

**Electronic supplementary material:**

The online version of this article (10.1186/s12885-019-5351-8) contains supplementary material, which is available to authorized users.

## Background

Delirium is the most common neuropsychiatric complication in patients with advanced cancer especially during hospitalization, with incidence rates ranging from 16 to 85%, depending on the stage of disease [1–7]. In a previous study from our group we found a higher number of patients with skin cancer and brain cancer in the group of patients with delirium compared to the patients without delirium [8]. Other studies showed conflicting results [9, 10]. Therefore, there is no compelling evidence that delirium is more prevalent in certain cancer types. Because attention and awareness deficits impede the ability to communicate and participate in treatment decisions and symptom assessment, delirium has a negative influence on quality of life in a crucial phase at the end of life [1]. The presentation of delirium is quite variable among patients, and even within a given patient because of its waxing and waning course [11]. This hampers recognition and adequate treatment of delirium [1]. Therefore it is recommended to screen for delirium in patients with (advanced) cancer admitted to the hospital [1, 11]. The diagnosis delirium should be made according to the DSM-criteria for delirium. Currently, version 5 is the most recent, but most screening and diagnostic instruments are based on the DSM-IV [12]. Efforts are being made to validate these instruments with the DSM 5 criteria [13, 14].

Available screening instruments which have been designed to be used by health care professionals for evaluating patients for possible delirium symptoms include: CAM [15], NEECHAM Confusion Scale [16], DOSS/DOS [17, 18], Nu-DESC [5], ICDSC [19], and PAED scale [20]. Some of these instruments have been designed to be used in a specific treatment setting like the ICU, whereas others focus on specific age groups, like children and adolescents. There is no specific screening instrument for delirium in patients with advanced cancer. A comparison between various features of the available screening and diagnostic instruments for delirium in adults was made by Grover and Kate in 2012 [12].

Delirium screening should be preferably performed by nurses because they have frequent contact with the patient throughout the day, and could therefore easily observe changes in the patient’s attention and awareness over time, which is one of the main criteria for delirium according to the DSM 5 criteria [21]. The Delirium Observation Scale (DOS) appears to be the most suitable nurse-rated screening instrument for patients in general medical and surgical wards with a strong foundation in the DSM-IV criteria and good psychometric properties [3, 22]. It can be assessed by nurses without specific training, and is experienced as user-friendly. A previous, small study by Detroyer et al suggests good sensitivity and specificity of the DOS in a palliative care population [23]. The aim of our study is to evaluate the diagnostic accuracy of the DOS as screening instrument for hospitalized patients diagnosed with advanced cancer.

## Methods

### Patients

Hospitalized patients with advanced cancer admitted to the medical oncology ward of six sites (1 university cancer center, 3 teaching hospitals, 2 high-care hospices) in the Netherlands were recruited between January 2011 through December 2015. Patients and/or their legal representatives were asked for informed consent to participate in this diagnostic study, as part of a randomized controlled trial (RCT), which compared the efficacy of haloperidol to olanzapine in case the patient was diagnosed with delirium (ClinicalTrials.gov identifier NCT01539733). The study was conducted according to Good Clinical Practice guidelines, the Declaration of Helsinki and local laws, and was approved by the institutional review boards of each participating study site.

Patients with any type of cancer in an advanced stage, of 18 years and older who were fluent in the Dutch language were considered eligible. Patients with pre-existing cognitive impairment (such as Alzheimer’s disease), or psychiatric comorbidity that might hamper delirium diagnosis (e.g. schizophrenia) were excluded from this study. Also, patients using antipsychotic or neuroleptic medication for other reasons than neuropathic pain management were excluded. Additional exclusion criteria were based on contra-indications for the use of haloperidol or olanzapine, like high risk at alcohol withdrawal delirium, glaucoma, Parkinson’s disease, QTc-interval prolongation > 500 msec at baseline ECG, a history of malignant neuroleptic syndrome and concomitant treatment with anti-convulsive drugs.

### Study assessments

Patients who were included in the trial were screened for delirium by their nurse using the DOS at the moment of admittance, and subsequently three times a day, biweekly during their stay in the hospital. Each DOS positive patient (DOS ≥3) was randomly matched with a DOS negative patient (DOS < 3) to evaluate the accuracy of the DOS. DOS positive patients and the randomly matched DOS negative patients were assessed with the Delirium Rating Scale-R^− 98^ (DRS-R-98) by a trained independent assessor, who was blinded with regard to the DOS score of a patient.

To prevent duplication bias patients were excluded from the DOS negative group if they were included in the randomised treatment part of the study during a later admission.

### DOS

The original version, the Delirium Observational Screening Scale (DOSS), consisted of a 25-item scale based on the *DSM-IV* criteria for delirium [17]. The scale was designed to capture early symptoms of delirium that nurses could observe during regular care. The scale was subsequently reduced to 13 observations, and is known as the Delirium Observation Scale (DOS) [18]. Each item can be rated as normal (score, 0) or abnormal (score, 1). A total score of 3 or more points indicates delirium. Completion of the instrument requires less than 5 min. A small descriptive study evaluated the DOS in patients admitted to a palliative care unit [23]. The DOS was compared to the CAM and the Delirium Index and showed good psychometric properties. Moreover, it was experienced as user-friendly by the bedside nurses. Internal consistency, predictive validity, and concurrent and construct validity of the DOS were tested in two prospective studies in high risk groups: geriatric medicine patients and elderly hip fracture patients [18]. The DOS has high internal consistency (0.96) and high content validity (α = 0.93). In these study groups the DOS scale had a sensitivity of 89–100% and a specificity of 68–88%. The positive predictive value was 47%, the negative predictive value was almost 100%. The DOS was also able to measure severity of delirium in geriatric patients [24]. Moreover, the DOS proved to be a good instrument to facilitate early recognition of delirium in patients who undergo cardiac surgery: the sensitivity and specificity of the DOS was 100 and 96.6% respectively [25].

### DRS-R-98

The DRS-R-98 is a revised version of the Delirium Rating Scale [26, 27]. The DRS-R-98 consists of 13 severity items that are scored from 0 (not present) to 3 points (severely present), and three diagnostic items, all of which are rated over the past 24 h. Severity scores range from 0 to 39, and total scores range from 0 to 46. The DRS-R-98 is designed to be completed by a trained professional and takes about 10 to 15 min to complete [27]. The DRS-R-98 has a high internal consistency (0.90) and when using a cut-off of 17.75 points on the total scale a sensitivity of 92% and specificity of 95% [27]. Inter-rater-consistency was high in the validation study by Trzepacz et al (0.99) [27]. The DRS-R-98 severity scale has the great benefit that it can be used for repeated measurements to assess the response to delirium treatment [27]. Recently, the DRS-R-98 has been validated for the new DSM-5 criteria [28]. The DRS-R-98 was chosen as a gold standard to evaluate the accuracy of the DOS because of its good psychometric qualities, because it has been validated in a palliative care setting, and is available in the Dutch language (the first language of the included patients and the researchers in this study group) [27].

### Statistics

A sample size calculation was conducted to determine an 80% power in demonstrating that the DOS has a sensitivity of at least 90%. A sample of 100 patients per group (DOS positive vs. DOS negative) was needed when the sensitivity of the DOS was assumed to be 95% [18, 25].

Primary endpoints to assess the accuracy of the DOS are the sensitivity and specificity of the DOS score compared with the DRS-R-98 as the gold standard. Sensitivity, specificity, negative and positive predictive value are reported with 95% confidence intervals, calculated with the Wilson method [29]. Analyses will be corrected for partial verification, because only a proportion of DOS negative patients admitted to the study sites were included in this study. For the baseline characteristics standard descriptive statistics were used. Statistical analyses were performed with IBM SPSS version 22.0 (IBM, Armonk, NY, USA).

## Results

**Fig. 1:**
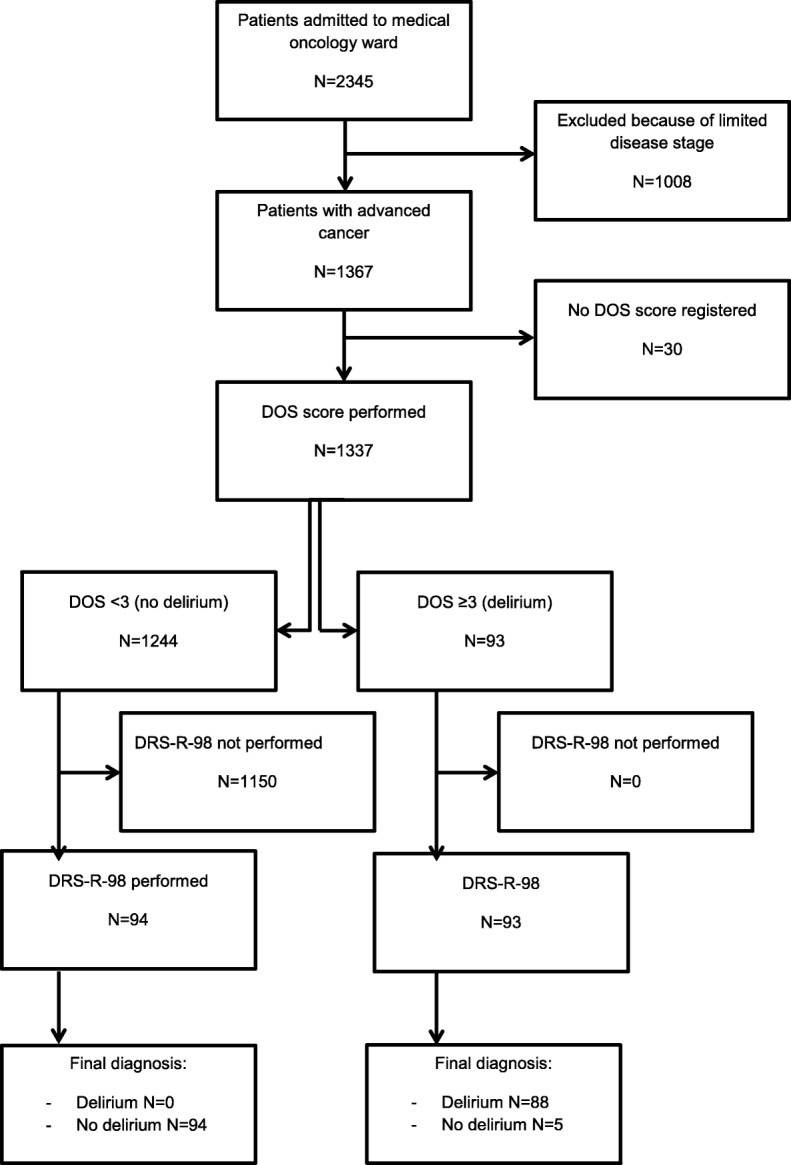
Study flow chart

Between January 2010 and January 2016, 100 consecutive DOS negative and 95 DOS positive patients were included in the study. One DOS positive patient withdrew consent for participating in the study while recovering from delirium. Four DOS negative patients were included in the DOS positive group during a later hospital admission. These patients were excluded from the DOS negative group during the analyses. For one patient in each group all data were missing, leading to a total of 94 DOS negative and 93 DOS positive patients (Fig. 1). The demographic and clinical characteristics are described in Table 1. The mean (SD) age was 68 (11) in the DOS positive group and 60 (13) in the DOS negative group (*p* < 0.001). Other characteristics obtained at baseline were not significantly different between the groups. None of the patients in the DOS negative group were diagnosed with delirium. Eighty-eight of the patients in the DOS positive group were diagnosed with delirium, see Table 2. The sensitivity and specificity of the DOS were > 99.9% (95%CI: 95.8–100.0%) and 99.6.% (95%CI: 95.5–100.0%) respectively, the negative predictive value (NPV) was 94.6 (95%CI, 88.0–97.7%), and the positive predictive value (PPV) was > 99.9% (95%CI, 96.1–100.0%), see Table 3.

**Table 1:** Baseline characteristics

	DOS negative *N* = 94 (SD or %)	DOS positive *N* = 93 (SD or %)	*p*
Age	60 (12.9)	68 (11.1)	<.001
Gender			
Male	58 (62%)	66 (71%)	
Female	36 (38%)	27 (29%)	.180
Tumor type			
Gastro-intestinal	40 (43%)	28 (30%)	
Breast	6 (6%)	6 (7%)	
Genito-urethral	23 (25%)	29 (32%)	
Skin	4 (4%)	8 (9%)	
Lung	0	3 (3%)	
Head&neck	5 (5%)	2 (2%)	
Brain	0	2 (2%)	
Sarcoma	4 (4%)	1 (1%)	
Hematological	1 (1%)	9 (10%)	
Other	11 (12%)	5 (5%)	.484
Brain metastasis			
No	89 (95%)	87 (94%)	
Yes	5 (5%)	6 (6%)	.767

Baseline characteristics of the patients in this study. No statistically significant differences were found except for age; Delirium Observational Scale (DOS) positive patients, who screened positive for delirium with a score ≥ 3 on this scale, tended to be older than DOS negative patients

**Table 2:** DOS vs. DRS-R-98

	DRS-R-98 no delirium (< 17.75)	DRS-R-98 delirium (≥17.75)	Total
DOS no delirium (< 3)	94	0	94
DOS delirium (≥3)	5	88	93
Total	99	88	

Distribution of the results of the DOS and DRS-R-98 scores

**Table 3:** Accuracy of the DOS

	Accuracy of the DOS	Wilson Score 95%CI lower limit	Wilson Score 95%CI upper limit
Sensitivity	1	0.958184	1.000004
Specificity	0.995997	0.955293	0.99966
PPV	0.946237	0.880258	0.976826
NPV	1	0.960748	1.000004

Sensitivity, specificity, positive predictive value (PPV) and negative predictive value (NPV) of the DOS, with 95% confidence intervals (95% CI) calculated by the Wilson method

The median DOS score of the DOS positive patients was 4.0 (IQR 4.0–6.0). Higher DOS scores correlated with higher DRS-R-98 total scores, see Table 4.

**Table 4:** DRS scores in DOS positive patients

DOS score	N=	DRS-R-98 total score median	DRS-R-98 total score IQR
3-4^a^	50	22.3	19.0–24.1
> 4-6^b^	23	23.5	21.5–26.0
>6^c^	20	26.0	21.8–27.4

Distribution of the DRS-R-98 scores in patients screened positive for delirium with the DOS. When comparing the absolute DOS score to the DRS-R-98 total score a linear association was found; B 0.677, 95% CI .229–1.126, P0.004

^a^1st and 2nd quartile

^b^3rd quartile

^c^4th quartile

Four of the five patients with a DOS of 3 or higher who were not confirmed to be delirious by the DRS-R-98 score (score < 17.75), had DRS-R-98 total scores > 12 points. The latter cut-off has been used as a more inclusive cut-off for delirium in some studies [30, 31]. One patient scored being delirious on the DOS but only scored three points on the DRS-R-98. We speculate that he/she may have displayed only temporarily signs of delirium which had resolved by the time the DRS-R-98 was conducted.

## Discussion

To our knowledge this is the first study to evaluate the accuracy of the DOS in a large group of patients with advanced cancer. Our data showed that the DOS is a very sensitive and specific instrument to screen for delirium in hospitalized patients with advanced cancer. It might also give an impression of the delirium severity.

Compared to other screening instruments, such as the CAM [15], NEECHAM Confusion Scale [16], and the Nu-DESC [5], the DOS has shown better sensitivity and specificity. It has the benefits that it is quick to administer, and does not require training [12].

Strengths of the study are that we were able to include a large group of patients, and that the DRS-R-98 assessment was performed by an independent assessor who was blinded to the DOS score of the patient. Also, the nursing staff was already used to complete the DOS scale during routine care, so we did not need a run-in or training period for the study. In addition, during the conduct of the study the nurses gave very positive spontaneous feedback on the effort it took to complete this questionnaire.

There are several limitations to this study. First, the observed incidence of delirium at the study sites was low [8]. Therefore, it was necessary to adjust the results for non-verification. Even after this adjustment, we have convincing results that the DOS is an accurate screening instrument for delirium screening in patients with advanced cancer. Second, while other baseline characteristics were evenly distributed over the DOS positive and DOS negative patients, patients who were DOS negative were younger than the DOS positive patients. This is concordant with the fact that advanced age is one of the known risk factors for the development of delirium [2]. Using age-matched comparators would however hamper the applicability of this study to the whole population of hospitalized patients with advanced cancer.

For this study we used a DRS-R-98 score of > 17.75 as a cut-off for delirium. The patients who screened positive for delirium with the DOS scale but not with the DRS-R-98, had (except for one) DRS-R-98 scores > 12, which has been classified as subsyndromal delirium in previous studies [30, 31]. One might argue that it might be beneficial for these patients as well to be treated for their symptoms.

## Conclusions

The DOS is an accurate instrument for detection of delirium in a population of hospitalized patients with advanced cancer. The DOS can be completed by nurses based on the observations made during regular nursing care, which makes it easily applicable as a screening tool for delirium in patients with advanced cancer. By early recognition and adequate treatment of this distressing delirium syndrome the quality of life of patients with advanced cancer can be improved.

## Additional file

Additional file 1: Study sites. (DOCX 11 kb)

## Abbreviations

95%-CI: 95% confidence interval
CAM: Confusion assessment method
DOS: Delirium Observation Scale
DOSS: Delirium observation screening scale
DSM: Diagnostic statistical manual
ECG: Electro Cardio Graphy
ICDSC: Intensive care delirium screening checklist
ICU: Intensive care unit
IQR: Inter quartile range
NEECHAM: Neelon Champagne Confusion Scale
NPV: Negative predictive value
Nu-DESC: Nursing Delirium Screening Scale
PAED: Pediatric anesthesia emergence delirium scale
PPV: Positive predictive value
RCT: Randomized controlled trial
SD: Standard deviation from the mean
