# Supplementary material for: Accuracy of the Delirium Observational Screening Scale (DOS) as a screening tool for delirium in patients with advanced cancer
Source: BMC Cancer. 2019 Feb 19;19:160. doi: 10.1186/s12885-019-5351-8 (PMC6379928; doi:10.1186/s12885-019-5351-8)
Supplement: Supplementary file 1 — Study sites. (DOCX 11 kb) [file 12885_2019_5351_MOESM1_ESM.docx]

Study sites

Amsterdam UMC, Vrije Universiteit, Amsterdam, department of medical oncology

Academic Hospice Demeter, De Bilt, The Netherlands

Hospice Kuria, Amsterdam, The Netherlands

Rijnstate Hospital, Arnhem, the Netherlands, department of Internal Medicine

Spaarne Gasthuis, Hoofddorp, the Netherlands, department of Internal Medicine
